# Identifying Gaps and Challenges in Acute Hepatitis B Surveillance in the Country of Georgia: Comprehensive Surveillance System Evaluation

**DOI:** 10.2196/72888

**Published:** 2025-12-01

**Authors:** Lika Karichashvili, Ketevan Galdavadze, Khatuna Zakhashvili, Maia Tsereteli, Ekaterine Ruadze, Sophia Surguladze, Pawel Stefanoff

**Affiliations:** 1 National Center for Disease Control and Public Health, Kakheti Highway N99, Tbilisi, 0198, Georgia, 995 592100979; 2 Mediterranean and Black Sea Programme in Intervention Epidemiology Training (MediPIET), European Centre for Disease Prevention and Control (ECDC), Stockholm, Sweden; 3 Integral Global Health, Tbilisi, Georgia

**Keywords:** acute hepatitis B, country of Georgia, evaluation, surveillance system, HBV

## Abstract

**Background:**

In 2012, the country of Georgia established an electronic integrated disease surveillance system (EIDSS) for acute hepatitis B virus (HBV) infection. All medical facilities must report suspected and confirmed acute HBV cases to the regional public health centers within 24 hours, which are subsequently registered in the EIDSS.

**Objective:**

This study aims to evaluate the acute hepatitis B surveillance system in Georgia in order to identify areas for improvement and develop recommendations that enhance its capacity to inform prevention and response efforts, supporting the elimination of viral hepatitis.

**Methods:**

For the evaluation of the acute HBV surveillance system from 2015 to 2020, we used the US Centers for Disease Control and Prevention updated guidelines. We assessed data quality by calculating the percentage of missing values for key variables. We assessed simplicity, acceptability, and flexibility by describing surveillance processes and by surveying public health center epidemiologists. We evaluated representativeness by comparing cases registered in EIDSS with cases registered in hospital discharges. We assessed timeliness by calculating the number of days from the date of diagnosis to the date of notification in EIDSS. We calculated the positive predictive value as the proportion of cases notified during 2018‐2020 having documentation of confirmatory tests in their medical records, meeting the confirmed case definition.

**Results:**

During 2015‐2020, 270 cases of acute viral hepatitis B were reported to the EIDSS. All notified cases were hepatitis B surface antigen positive. However, only 10 of the 19 (53%) key variables were complete. Hepatitis B test results were missing in most reported cases, despite 82% (223/270) being classified as “confirmed.” Simplicity and acceptability of the system were affected by 30% (31/104) of the respondents experiencing challenges with the EIDSS reporting form. The system had limited flexibility due to cumbersome procedures to implement any changes. Representativeness was limited, as only 41% (270/657) of confirmed cases recorded in the hospital discharge database were reported to the EIDSS. The average notification delay was 72 hours. Among 104 cases notified in 2018‐2020, 66 met the case definition, leading to a positive predictive value of 63%.

**Conclusions:**

The surveillance system for acute HBV infection was timely, although only 51% (139/270) of the cases were reported within the 24-hour notification target. The system was not representative and did not correctly ascertain cases. We recommend reconsidering the statutory notification time of 24 hours, revising notification forms and providing clear guidelines for data entry, and reporting all test results needed for adequate case classification to enhance data completeness and reliability of case classification.

## Introduction

### Hepatitis B as a Global Health Challenge

Hepatitis B is a potentially life-threatening liver infection caused by the hepatitis B virus (HBV). It can cause acute and chronic disease and increases the risk of death from cirrhosis and liver cancer [1]. Hepatitis B is transmitted by exposure to contaminated blood (injection drug use, unsafe medical injections, unscreened blood and blood products) and body fluids (perinatal, sexual transmission). The incubation period for HBV ranges from 2 to 6 months [2]. Laboratory tests including IgM to hepatitis B core antibodies (IgM anti-HBc) and hepatitis B surface antigen (HBsAg) are needed to confirm acute HBV infection and differentiate acute from chronic infections. Diagnosis of acute hepatitis B is more common in adults than in children, who remain mostly asymptomatic following exposure and have a high risk of progressing to chronic HBV infection [2]. Hepatitis B is preventable by vaccination, and the World Health Organization recommends 3 doses of hepatitis B vaccine for all children, health care workers, and persons at high risk of HBV infection [3].

### The Role of Viral Hepatitis Surveillance

Viral hepatitis surveillance is essential to detect outbreaks, monitor disease trends, identify risk factors for recent infections, estimate the burden of viral hepatitis and its sequelae, and assess the impact of vaccination and other preventive services. Surveillance for acute viral hepatitis through reporting and investigation of patients with symptoms of acute hepatitis presenting to health care facilities can help detect outbreaks and identify leading risk factors for infection, which informs the development of prevention and response activities. The prevalence of chronic HBV infection is mainly assessed through nationally representative serological surveys, which can monitor disease trends over time [2].

### Hepatitis B in Georgia

Georgia implemented both acute hepatitis B surveillance and nationally representative hepatitis B serosurveys [4]. In 2015 and 2021, the Georgian National Centre for Disease Control and Public Health (NCDC) and the US Centers for Disease Control and Prevention (CDC) conducted 2 serosurveys throughout the country to determine the burden of viral hepatitis. The results showed that in 2015 the prevalence of HBsAg was 2.8% in adults. By 2021, the prevalence of HBsAg in adults and children was 2.7% and 0.03%, respectively, indicating the need to scale up the prevention, screening, and treatment of hepatitis B in adults [4].

### Acute Hepatitis B Notification and Reporting System in Georgia

In 2005, Georgia included acute hepatitis B in the list of notifiable diseases, and health care providers were mandated to report cases to the national surveillance system [5]. The acute hepatitis B surveillance system is comprehensive and is designed to cover the entire country, including all hospitals nationwide. Since 2012, reporting of acute hepatitis B cases is done using electronic case reporting forms in the electronic integrated disease surveillance system (EIDSS). EIDSS is directly linked to the Georgian civil registry, meaning that the national unique identification number (ID) and date of birth in the notification section are automatically identified. This guarantees the accuracy of the personal ID number belonging to the notified case. If the case subject does not have a personal ID number (immigrant or refugee), they cannot be linked to the Georgian civil registry. In such situations, the epidemiologist is required to register the case in EIDSS by noting the passport number in the annotation. In addition, reporting of confirmed acute cases of hepatitis B is done using an electronic module of registration of discharged inpatients (IV-066). This is a separate register that collects the aggregated number of medical records from all patients discharged from hospitals across the country by the end of each month.

### Evaluating the Acute Hepatitis B Surveillance System

The World Health Organization recommends assessing viral hepatitis surveillance to identify potential barriers that might impact data quality and influence the use of data for action [2]. Surveillance of acute hepatitis B in Georgia has never been evaluated. We conducted an evaluation of the acute hepatitis B surveillance system in Georgia to identify areas for improvement and provide recommendations to ensure surveillance can inform prevention and response efforts and advance the elimination of viral hepatitis.

## Methods

### Description of the Surveillance System

Suspected cases of acute hepatitis B are usually identified in outpatient and inpatient facilities or private laboratories. The physician and laboratory personnel requesting the diagnostic tests are responsible for providing the patient with feedback regarding their test results. The physician is required to report each suspected or confirmed acute hepatitis B case within 24 hours to the municipal or regional primary health care center (PHC). Subsequently, within 72 hours of receiving the notification, the PHC epidemiologist starts a case investigation to collect relevant epidemiological data and enters the collected information in the EIDSS.

The EIDSS case reporting form is part of the public health surveillance system and contains 3 sections: (1) notification of data, (2) case investigation, and (3) laboratory results (Table 1). The notification section includes general demographic information (personal ID number, age, date of birth, sex, current residence, permanent residence, telephone number), symptom history, and the primary diagnosis. The case investigation section includes details of patient symptoms, exposures, hospitalization, vaccination history, information about contact, and final case classification. The laboratory investigation section includes test results.

**Table 1.** Description of variables in the electronic integrated disease surveillance system (EIDSS) case reporting form, Georgia, 2022.

Section	Subsection	Description of contents
Notification	Demographic information	Personal ID number, age, date of birth, sex, current residence, permanent residence, telephone number (If not residing in Georgia, records the country of residence)
Notification	General information	Date of completion of the paper form, reporting institution and receiving institution information, as well as investigator, investigator organization, initial date of investigation
Notification	Clinical information	Date of symptoms onset, status of the patient at the time of notification (alive or dead), initial diagnosis (if known), date of diagnosis and current location of the patient (hospital or house)
Notification	Note	Epidemiologists used this space for any additional comments. Additionally, if the case was not a resident of Georgia, epidemiologists entered the passport ID here
Case investigation	Clinical information	Initial case classification, date of exposure, date of symptoms onset, location of exposure, facility where patient first sought care, date patient first sought care, nonreported diagnosis from facility where patient first sought care, hospitalization, date of hospitalization, and antibiotic or antiviral therapy administered before specimen collection
Case investigation	Samples collection	Specimen type, date of reception or registration
Case investigation	Contact list	Demographic information of the patient’s close contacts
Case investigation	Case clinical classification	Clinical symptoms selected from a general list composed of all the symptoms characteristic of 74 communicable diseases
Case investigation	Epidemiological links and risk factors	Hepatitis B immunization history, risk factors and source of infection, information on medical manipulations and safety practices for potential sources of infection
Case investigation	Final case classification and outcome	Final case classification, final diagnosis, link to an outbreak, outcome, and additional comments
Laboratory results	Tests	Results of diagnostic tests, selected from a list

### Data Sources

All epidemiologists working at municipal, regional, and central levels have access to the EIDSS. There is a web version of EIDSS accessible from any device connected to the internet. Each epidemiologist has an individual username and password. The web version and analytical module are available in 3 languages (Georgian, English, Russian). Furthermore, epidemiologists at the NCDC have access to the EIDSS analytical module, allowing further analysis and preparation of annual surveillance reports.

Another source of data on confirmed acute hepatitis B cases is the hospital discharge form IV-066, which collects information from 265 hospitals across all 11 regions of Georgia. Each hospital is required to submit the list of discharge records to the NCDC’s statistics department no later than the 10th day of the month following the discharge of the patient. The IV-066 form consists of patient demographics, facility information, medical chart number, and confirmed diagnosis. The IV-066 database is linked to and validated against the Ministry of Health’s database, which is used to determine financial reimbursements to health care facilities. The database is strictly monitored by the Ministry of Health to ensure that diagnoses are correctly reported.

### Case Definitions

During 2015‐2020, the following case classifications for acute hepatitis B were used in Georgia:

- Suspected case: A person who has the following signs and symptoms: fever, headache, weakness, thirst, nausea, vomiting, diarrhea, abdominal pain, and one of the following: jaundice or a serum alanine aminotransferase level >100 IU/L—or in the absence of clinical signs, if there is a documented negative HBsAg test (HbsAg, “e” antigen—HbeAg or HBV nucleic acid testing) within 6 months of receiving a positive test result, the case was considered a suspected acute viral hepatitis B.
- Confirmed case: A suspected case having both IgM anti-HBc and HBsAg positive.

### Analysis of Surveillance Attributes

We restricted the evaluation of the acute hepatitis B surveillance system to the period from January 1, 2015, to December 31, 2020, as the case definition for acute hepatitis B was revised in January 2015.

To assess the acute HBV surveillance system, we used the CDC updated guideline “Updated Guidelines for Evaluating Public Health Surveillance Systems” [6]. We considered the following system attributes: flexibility, simplicity, acceptability, data quality, representativeness, timeliness, and positive predictive value (PPV).

### Flexibility

We assessed the flexibility of the EIDSS by describing the procedures required to adapt the system to new requirements, including the process for modifying questionnaires.

### Simplicity and Acceptability

To assess the simplicity and acceptability of EIDSS, during March-August 2022, we distributed an online questionnaire (Multimedia Appendix 1) to epidemiologists working across 59 PHCs in 10 regions and 2 out of 9 regional branches of the NCDC. We did not estimate a sample size due to the qualitative nature of the survey and attempted to include all PHC epidemiologists. We allowed the respondents 2 months to complete the questionnaire. During this period, we called and reminded them once every 2 weeks and sent a reminder email once a month.

### Data Quality and Representativeness

We assessed data quality in EIDSS by calculating the percentage of complete information for selected variables (demographic, laboratory, and case classification).

We evaluated representativeness by comparing the number of hospitalized cases recorded in the IV-066 database and those notified to EIDSS by year and region, and calculated reporting ratios by dividing cases registered in EIDSS by cases registered in IV-066. We also calculated the incidence of cases recorded in the EIDSS and IV-066 databases. Even though the IV-066 database has never been evaluated, for this calculation, we assumed that cases registered in the IV-066 database reflect HBV infections correctly diagnosed among hospitalized patients, as the database is linked to and validated against the Ministry of Health’s reimbursement system and is strictly monitored to ensure that diagnoses are correctly reported.

### Timeliness

To assess timeliness, we calculated delays between diagnosis and notification (target: maximum 24 h) and between notification and the start of the epidemiological investigation (target: maximum 72 h). The information on the start time of the epidemiological investigation is recorded by the PHC epidemiologists directly in the EIDSS during the case investigation process.

### Positive Predictive Value

To assess the PPV, we reviewed medical histories of confirmed acute hepatitis B cases registered in EIDSS during 2018‐2020. This period was selected because recent patient documentation was available in the hospital archives. We compiled a list of confirmed acute hepatitis B cases registered in the EIDSS during 2018‐2020 based on the location of hospitalization, and if this information was missing, based on the region of residence. We distributed the list to hospitals or regional PHCs according to the patient’s region of residence and hospital information and asked them to complete the following information based on review of medical records: date of birth, age, place of residence, date of notification, date when the case was registered in EIDSS, date of onset of symptoms, date of the patient’s first visit to the first care facility, name of the medical facility, whether or not there was hospitalization, and if so the date of hospitalization and date of discharge, date of final diagnosis, presence of clinical signs, IgM anti-HBc and HBsAg laboratory test results, outcome (recovered, died, or unknown), and final case classification. Then, we calculated PPV as the proportion of cases reported to EIDSS that met the criteria of a confirmed acute hepatitis B case.

### Ethical Considerations

The survey of regional epidemiologists who reported on their experience with EIDSS and notification was determined by the US CDC to represent a nonresearch public health activity and, therefore, did not require the CDC’s institutional review board (IRB) review. The protocol was approved by the NCDC IRB (registration number for the IRB decision letter: IRB #2022‐027). The study used routinely collected secondary surveillance data, and no personal or identifiable information was accessed. The questionnaire administered to regional epidemiologists was anonymous. The survey link was distributed to contact persons in each regional PHC together with a description of the survey’s purpose, intended data use, and assurance of anonymity. Participation required confirming consent on the survey landing page before proceeding. No IP addresses or other identifiers were collected. The NCDC had no influence over how the link was shared within regional departments, and it was assumed that only individuals who consented chose to participate. No incentives or compensation were provided.

## Results

During 2015‐2020, 270 suspected or confirmed cases of acute hepatitis B were reported to EIDSS, while 657 confirmed cases were reported in the hospital discharge registries (IV-066). A total of 104 regional epidemiologists and PHC employees responded to the survey. Most respondents (85%, 88/104) were female, and 79% (82/104) were epidemiologists.

### Analysis of Surveillance Attributes

#### Flexibility

To incorporate changes to information collected in EIDSS, NCDC’s epidemiologists need to submit an Excel form with all proposed changes. Their proposed changes need to be approved by the head of the communicable diseases department. After approval, the list of approved changes is sent by mail to the developer of the EIDSS database. Any changes to the form usually take 2‐3 months. Following each major change, PHC epidemiologists need to be trained on the new changes.

#### Simplicity and Acceptability

Of 104 survey respondents, 30% (31/104) found that the EIDSS case reporting form was difficult to complete. Most respondents (65/104, 62%) believed that information on “safe practices” in health facilities was obsolete as municipal public health services do not have the mandate to evaluate infection prevention and control measures in medical facilities. Only 45% (47/104) of the respondents reported sufficient knowledge of an acute hepatitis B investigation, including details needed to obtain from the patient’s medical documentation and during case interviews. Of the 33 respondents who were not able to thoroughly investigate the notified cases, 16 (48%) could not access relevant laboratory results. The predefined list of tests included in the EIDSS case investigation form does not include the IgM anti-HBc for acute hepatitis B, required for the confirmed case ascertainment. If the PHC epidemiologists obtain the laboratory test results, they can enter this information in the free-text “Note” field. However, this is not a standardized data entry.

#### Data Quality

While demographic information such as sex, age, and place of residence was recorded with completeness rates of 96%, 99%, and 100%, respectively, critical variables, such as hepatitis B vaccination date and laboratory test results, were entirely missing. Reasons for missing hepatitis B vaccinations were noted in only 21% (58/270) of the cases. While 82% (223/270) of the cases were classified as “confirmed,” this was often based only on HBsAg positivity without results of IgM anti-HBc, undermining the accuracy of this classification (Table 2).

**Table 2.** Completeness of key variables, in the acute hepatitis B case reporting form submitted to the electronic integrated disease surveillance system, Georgia, 2015‐2020 (N=270).

Variable	Values, n (%)
Sex	260 (96)
Age	267 (99)
Place of residence	270 (100)
Hospitalized	231 (86)
Outpatients	17 (6)
Information about last vaccination date	0 (0)
Reason why hepatitis B vaccination was not given	58 (21)
Laboratory test (HBsAg^a^, IgM anti-HBc^b^, HBeAg^c^)	0 (0)
Final classification “probable”	5 (2)
Final classification “possible/suspicious” “lost from supervision” " rejected”	8 (3)
Final classification “confirmed”	223 (82)

a HBsAg: hepatitis B surface antigen.

b IgM anti-HBc: immunoglobulin M antibody to hepatitis B core antigen.

c HBeAg: hepatitis B e antigen.

### Representativeness

Based on the review of hospital discharge forms (IV-066), during 2015‐2020, 657 confirmed cases of acute hepatitis B were diagnosed in hospitals, of which 270 (41%) were registered in EIDSS. The incidence of cases recorded in hospital discharges (IV-066) varied between 2 and 4 cases per 100,000 inhabitants. In 2015, the incidence was 2 per 100,000, reaching its peak at 4 per 100,000 in 2017. Subsequently, the incidence slightly declined to 3 per 100,000 in 2018 and 2019, and further dropped to 2 per 100,000 in 2020. As for the EIDSS data, incidence remained constant at 1 case per 100,000 each. This comparison suggests that EIDSS reporting did not capture the trend in incidence of suspected acute hepatitis B that was observed in hospital discharge forms.

### Timeliness

The average delay between diagnosis and notification to the PHCs was 72 hours, while the national guideline required notification within 24 hours. Almost half (139/270, 51%) of the reported acute HBV cases were notified within 24 hours. Furthermore, on average, there was a 48-hour time lapse between case notification and the beginning of the epidemiological investigation, which falls within the national guideline that mandates the investigation to begin within 72 hours of receiving notification.

### Positive Predictive Value

Medical chart reviews were used to validate 104 patients who were registered as confirmed cases of acute hepatitis B during 2018‐2020. Of these, 39 (37.5%) lacked sufficient information on clinical or laboratory data to determine if the patient was a confirmed case of acute hepatitis B.

Thus, we estimated the PPV at 63%, since only 66 cases met the criteria for a confirmed case. All confirmed acute HBV cases presented with jaundice and dark urine. Their average age was 36(SD 14.3) years, and 44 (66%) were men. Most (53/67, 79%) resided in Tbilisi (Table 3). All true positive cases were hospitalized. The average alanine aminotransferase level for these 66 true positive cases was 2313 IU/L (SD 1094). HBV DNA testing was performed in 46 out of 66 cases (Table 3).

**Table 3.** Demographic and clinical characteristics of confirmed (true positive) acute hepatitis B cases in Georgia, 2018‐2020 (N=67).

Variable	Values	
Gender, n (%)		
Men	44 (66)	
Women	22 (33)	
Region of residency, n (%)		
Guria	1 (2)	
Ajara	12 (18)	
Tbilisi	53 (79)	
Symptoms, n (%)		
Fever	27 (41)	
Stomach ache	37 (56)	
Nausea	53 (80)	
Lack of appetite	63 (95)	
Weakness	64 (97)	
Fatigue	65 (98)	
Jaundice	66 (100)	
Dark urine	66 (100)	
Mean age (y) (SD)	36 (14.3)	
Mean ALT^a^ level (IU/L) (SD)	2313 (1094)	

a ALT: alanine aminotransferase.

## Discussion

### Principal Findings

The comprehensive evaluation of the acute hepatitis B surveillance system in Georgia revealed areas of improvement. Georgia’s acute hepatitis B surveillance reporting in EIDSS was timely, as 51% (139/270) of the acute hepatitis B cases were reported within 24 hours, as required by national guidelines. However, the system was not sufficiently representative and did not correctly ascertain cases.

While Georgia’s system adapted to the new requirements, implementing changes is complicated and takes time (2‐3 mo). Accelerating adaptation timelines would enhance the Georgian system’s flexibility.

The simplicity of Georgia’s EIDSS case reporting system was another area of concern. Although the reporting form for acute hepatitis B aimed to collect comprehensive data on transmission modes, risk factors, and contacts, public health specialists lacked access to key required information, which resulted in incomplete forms; this limited the utility of the epidemiological investigation section at the national level. Additionally, local epidemiologists reported technical difficulties collecting and entering key variables like IgM anti-HBc results into the EIDSS. Without access to laboratory data, PHC epidemiologists often submitted incomplete notifications, complicating case classification at the national level, which led to a decreased PPV of 63%. This highlighted the importance of reporting all test results to enable accurate case classification and public health action. Additionally, variables such as the date of last hepatitis B vaccination are crucial for assessing vaccine effectiveness and identifying potential cases of vaccine failure, which are essential for guiding immunization policy and response strategies.

Our survey of regional epidemiologists found that only 45% (47/104) had sufficient knowledge of acute hepatitis B investigation, leading to potential misclassification of cases and reduced validity of notifications. These findings emphasize the need for further training at the regional and health facility levels to improve the understanding of case definitions, investigation methods, and data reporting to EIDSS.

Incomplete data was an issue, with important fields such as vaccination status and the source of infection often left blank. Improving data completeness in Georgia is essential for ensuring the accuracy and reliability of the surveillance system. Underreporting undermined the representativeness of the system. The overall reporting ratio was 41%, with significant variation across regions. Discrepancies between the number of suspected cases reported to EIDSS and those recorded in the hospital discharge database highlighted the need for cross-referencing data from multiple systems to ensure the representativeness and completeness of case reporting.

The survey of regional epidemiologists may not reflect the experience of all surveillance users. We selected a convenience sample of employees of PHCs and collected mostly qualitative feedback. To address this potential shortcoming, we tried to contact all PHCs and send several reminders to include as many eligible respondents as possible. In addition, a substudy validating the classification of acute hepatitis B cases utilized medical histories from reported cases between 2018 and 2020. This period did not cover the full period of our investigation. However, as we obtained complete information on these cases, we believe our results reflect true case classification practices. Access to diagnostic testing is another aspect of surveillance that can affect the reliability of surveillance notifications. Although we did not investigate access to diagnosis, it is generally recommended to be performed in all regional hospitals. The completeness of data relies heavily on the accuracy of entries by epidemiologists. If there are inconsistencies or gaps in data entry, the assessment may underestimate or overestimate the actual completeness. The assumption that all hospitalizations in IV-066 are correctly classified as acute HBV infections might introduce errors. Misclassifications or omissions in either database (IV-066 or EIDSS) may affect the accuracy of the representativeness analysis, leading to an under- or overestimation of reporting ratios. Additionally, since IV-066 includes only patients who have been admitted to hospitals, our comparison was limited to hospitalized cases in both the IV-066 and EIDSS databases to ensure consistency and accuracy in the representativeness analysis.

### Comparison With Similar Studies

Similar evaluations were performed in China [7], Armenia [8], Germany [9], and South Korea [10]. Similar to Georgia, China’s hepatitis B surveillance system, particularly in Fujian, Hainan, and Gansu provinces, relied on case reporting through the National Notifiable Disease Reporting System [7]. The system depended on clinicians to report hepatitis B cases and classified them as acute or chronic, according to national guidelines, as it does in Georgia. Both Georgia and China had electronically integrated surveillance data, allowing public health authorities to gather, monitor, and analyze data efficiently, providing near real-time data access. Both China and Georgia faced challenges with misclassification and data quality in hepatitis B surveillance. While China’s system demonstrated problems with underreporting and inconsistent testing practices, Georgia also had data quality issues, especially with incomplete laboratory confirmations and misclassification of cases based on HBsAg without IgM anti-HBc.

Both Georgia’s and South Korea’s hepatitis B surveillance systems focused on acute cases. South Korea, which transitioned to mandatory reporting in 2011, demonstrated a high positive predictive value (94.4%) and good data quality [10]. However, it faced challenges with chronic cases being misreported as acute, a misclassification also identified in Georgia. Unlike South Korea, Georgia faced more significant challenges in data completeness and misclassification.

Similar to Georgia, Armenia’s surveillance systems reflected the legacy challenge from the Soviet public health framework. Armenia’s system, modeled on the Soviet-era framework, was extensive and involved reporting at various administrative levels [8]. It collected both aggregated and case-based data for numerous infectious diseases. It lacked complex procedures and standardized case definitions. Georgia, on the other hand, struggled with incomplete reporting of key variables and regional underreporting.

Both Germany and Georgia faced challenges with data completeness, though the focus of these issues differed: Germany struggled with recording transmission routes and vaccination status, while Georgia faced gaps in laboratory results and vaccination information. Germany operated a mandatory surveillance system under the Protection Against Infection Act, requiring physicians and laboratories to report acute hepatitis B cases [9]. The system was well established with a clear data flow through local, state, and national public health authorities. Although Germany’s system performed well in terms of timeliness, it has experienced a decline in data quality over the 10-year evaluation period, especially in capturing transmission routes and vaccination history.

### Conclusions

Acute hepatitis B surveillance in Georgia suffers from unnecessarily complex investigation procedures. The complicated case reporting forms are time-consuming and cumbersome to complete. The users are not able to complete the information necessary for classifying cases. In particular, the information on confirmatory testing is not recorded reliably, leading to the misclassification of a large proportion of notified cases. In addition, a large proportion of regional epidemiologists are not aware of the notification procedures and case definition criteria. Despite these challenges, the system demonstrated positive results in terms of timeliness. This investigation provides essential insights into the strengths and weaknesses of the Georgian hepatitis B surveillance system, offering targeted recommendations to enhance data accuracy, improve case classification, and ultimately strengthen public health response capabilities.

### Recommendations

We recommend revising the EIDSS case reporting form to simplify data entry and ensure that only essential information for case classification is required, improving data completeness, simplicity of EIDSS, and acceptability. Additionally, to improve representativeness and PPV, we recommend developing clear guidelines on proper case notification and investigation procedures, particularly focusing on the use of confirmatory tests, disseminating to regional epidemiologists and reporting physicians, and providing easy-to-access training in notification procedures. We recommend conducting targeting training sessions for public health specialists to improve timeliness.

## Supplementary material

10.2196/72888 Multimedia Appendix 1 Study questionnaire.

## Abbreviations

CDC: US Centers for Disease Control and Prevention
EIDSS: electronic integrated disease surveillance system
HBsAg: hepatitis B surface antigen
HBV: hepatitis B virus
IgM anti-HBc: immunoglobulin M antibody to hepatitis B core antigen
IRB: institutional review board
NCDC: National Centre for Disease Control and Public Health
PHC: public health center
PPV: positive predictive value
