# Supplementary material for: Identifying Gaps and Challenges in Acute Hepatitis B Surveillance in the Country of Georgia: Comprehensive Surveillance System Evaluation
Source: JMIR Public Health Surveill. 2025 Dec 1;11:e72888. doi: 10.2196/72888 (PMC12670053; doi:10.2196/72888)
Supplement: Multimedia Appendix 1 [file publichealth-v11-e72888-s001.docx]

# **Appendix 1:** Survey of user experience with acute hepatitis B notifications.

**Questionnaire ID _____________________**

**Name of establishment** ------------------------------------------------------------------

1. **Sex:**
2. Female
3. Male
4. **Occupation**
5. Epidemiologist
6. Person responsible for immunization
7. Other --------------------------------
8. **Have you been trained in viral hepatitis surveillance?**
9. Yes
10. No (Skip to q6)
11. I don’t know (Skip to q6)
12. **When did you receive training in viral hepatitis surveillance?**
13. <1 year
14. 1-3 years ago
15. 3-5 years ago
16. **Who provided the training?**
17. NCDC representative
18. Infectious disease doctor
19. I did an independent online training
20. Other: __________________________
21. **Which of these cases are subjects to urgent notification (it is possible to mark several answers)?**
22. Possible/suspected case of acute hepatitis B.
23. Probable case of acute hepatitis B.
24. Confirmed case of acute hepatitis B.
25. Case of any classification of acute hepatitis B.
26. Case of chronic hepatitis B.
27. Acute hepatitis B is not subject to urgent notification
28. **Do you receive urgent notifications of acute hepatitis B cases (F. #58/1)?**
29. Yes
30. No (go to q15)
31. I don’t know (go to q15)
32. **From where do you get the acute hepatitis B notification from (it is possible to mark several answers)?**
33. From Clinics
34. From laboratories
35. From patients
36. Reporting an acute hepatitis B case is not mandatory
37. **On average how soon after receiving a diagnosis/laboratory test result do you receive notifications of acute hepatitis B?**
38. 12 hours
39. 24 hours
40. 72 hours
41. After a month
42. Other _______________________
43. **How often do you receive reports of acute hepatitis B from clinics / laboratories based only on a positive HBsAg test result?**
44. Always or most of the time
45. Occasionally
46. Rarely
47. Never
48. **How often do you receive reports of acute hepatitis B from clinics / laboratories based only on a positive IgM anti-HBc test result?**
49. Always or most of the time
50. Occasionally
51. Rarely
52. Never”.
53. **In what form do you get a notification of an acute hepatitis B case (it is possible to mark several answers)?**
54. E-mail
55. Phone
56. Printed form (form 58/1)
57. In various forms (Specify)_____________________________________
58. **When you receive report about acute HBV, on average how complete is the information reported in the case of acute hepatitis B (according to the standard case definition)?**
59. Complete (The notification includes complete information on the demographic, risk factor, clinical, and laboratory variables included in form 58/1)
60. Sufficient (The notification includes partial information on the demographic, clinical, and laboratory variables included in form 58/1)
61. Insufficient (The notification only demographic variables included in form 58/1)
62. **From how many clinics/laboratories did you receive report about acute HBV in the past 12 months (Specify exact number)?**
63. __________________________________________________
64. We didn’t receive notifications (Please indicate the reason)

_____________________________________________________

1. I don’t know/I don’t remember
2. **When did the registration of cases of acute hepatitis B in EIDSS start In Georgia?**

__________/_______

1. (mm/yy)
2. I don’t know/ I don’t remember
3. **How do you act in the time between receiving an acute hepatitis B case report before registering it in EIDSS? (it is possible to mark several answers)**
4. I collect case information in accordance with Form 58/1 variables
5. I record the received information in a 60/B journal paper form
6. I check case data in EIDSS to avoid duplication
7. All of the above
8. **When you receive urgent notification about acute hepatitis B, on average how soon do you start the epidemiological investigation of the case?**
9. Within 24 hours of notification
10. Within 48 hours of notification
11. Within 72 hours of notification
12. I do not think that time is important
13. **How do you conduct an epidemiological investigation for acute hepatitis B (it is possible to mark several answers)?**
14. I conduct a telephone interview with an infected person
15. I contact the institution that sent the message with the purpose of clarifying the information
16. I study the medical history of the case in detail at the clinic
17. I conduct an epidemiological investigation with the patient at home
18. I don’t conduct an epidemiological investigation
19. **What is most useful for acute hepatitis B epidemiological investigation:**

a) Gather information from illness history

- 1. Gather information by interviewing the patient
  2. Finding information in both of the abovementioned ways
  3. I do not know
  4. Other (specify)____________________________________________

1. **Do you think that you are doing a thorough epidemiological examination of an acute hepatitis B case (meaning checking information on all variables in the epidemiological questionnaire)?**
2. Yes (go to q22)
3. No
4. I don’t know
5. **In your opinion what is the reason why you are not always able to carry out a thorough epidemiological investigation of an acute hepatitis B case (it is possible to mark several answers)?**
6. Access to the medical history is restricted/limited
7. The medical history does not include all the information required by the epidemiological questionnaire
8. There is no complete diagnosis at the city/municipality level and patients are referred to another clinic
9. Due to the delayed notification, it is inconvenient to retrospectively interview the case
10. Other (Please specify) ____________________________________________________________
11. **What type of data knowledge is required to be granted "confirmed" status in a possible case of acute hepatitis B?**
    1. Existence of clinical signs of acute viral infection
    2. Alteration of liver enzymes
    3. The blood sample is HbsAg positive
    4. The blood sample is IgM anti-HBc positive
    5. None of the above
    6. All of the above
12. **Does the acute hepatitis B epidemiological questionnaire in EIDSS allow the case to be assigned a "possible/suspicious" or "probable" or "confirmed" status in the final classification?**
13. Only possible/suspicious
14. Only probable
15. Only confirmed
16. According to the provided information, any status can be granted
17. I don’t know
18. Other (specify)
19. **From your experience how difficult is it to complete the Acute Hepatitis B Epidemiological Questionnaire in EIDSS?**
20. Very Easy (go to q26)
21. Easy (go to q26)
22. Hard
23. Very hard
24. **From your experience which field/variable of the epidemiological questionnaire is difficult for you to fill?**
    1. About the reporting institution
    2. About case contacts
    3. About clinical symptoms
    4. About epidemiological links
    5. About risk factors
    6. About testing
    7. About safe practices
    8. All of the above
    9. I do not know
25. **How do you research information about "safe practices" during an acute hepatitis B case study in EIDSS?**
26. I research this in the facility notifying the case
27. I research it during an interview with a patient
28. I am not researching this issue
29. I do not know what is meant by this variable
30. Other (Specify)______________________________________________
31. **Do you think that the variables on "safe practice" should be removed from the questionnaire of the acute hepatitis B case study in EIDSS?**
32. Yes, I think they should be removed
33. No, I think they should stay
34. I do not know
35. **Do you have the necessary technical support (individual computer, constant internet, uninterruptible power supply, etc.) to work in EIDS?**
36. Yes
37. No
38. Partially (specify)______________________________________
39. **Does the Public Health Centre provide any feedback to the reporting institutions after the epidemiological investigation?**
40. Yes, Specify________________________
41. No
42. I don’t know
43. **Who do you consult for issues related to acute hepatitis B surveillance?**
44. From a NCDC epidemiologist
45. From a regional/LSS/ZDL epidemiologist
46. From an infectious disease specialist
47. Other (Specify)

----------------------------------------------------------

1. **Do you receive feedback from NCDC on acute hepatitis B surveillance?**
2. Yes
3. No
4. I don’t know
